# Australian state influenza notifications and school holiday closures in 2019

**DOI:** 10.12688/f1000research.21145.3

**Published:** 2021-05-26

**Authors:** Anna Mae Scott, Mina Bakhit, Justin Clark, Melanie Vermeulen, Mark Jones, David Looke, Chris Del Mar, Paul Glasziou

**Affiliations:** 1 Institute for Evidence-Based Healthcare, Bond University, Robina, Queensland, 4226, Australia; 2 Department of Medicine, University of Queensland, St Lucia, Queensland, 4072, Australia; 3 Infectious Disease and Clinical Microbiology, Princess Alexandra Hospital, Brisbane, Queensland, Australia

**Keywords:** Influenza, influenza epidemic, child, adolescent, school closure, school holidays, absenteeism

## Abstract

**Background**: The impact of school holidays on influenza rates has been sparsely documented in Australia. In 2019, the early winter influenza season coincided with mid-year school breaks, enabling us the unusual opportunity to examine how influenza incidence changed during school holiday closure dates.

**Methods**: The weekly influenza data from five Australian state and one territory health departments for the period of week 19 (mid-May) to week 39 (early October) 2019 were compared to each state’s public-school holiday closure dates. We used segmented regression to model the weekly counts and a negative binomial distribution to account for overdispersion due to autocorrelation. The models’ goodness-of-fit was assessed by plots of observed versus expected counts, plots of residuals versus predicted values, and Pearson’s Chi-square test. The main exposure was the July two-week school holiday period, using a lag of one week. The effect is estimated as a percent change in incidence level, and in slope.

**Results**: School holidays were associated with significant declines in influenza incidence in three states and one territory by between 41% and 65%. Two states did not show evidence of declines although one of those states had already passed its peak by the time of the school holidays. The models showed acceptable goodness-of-fit. The first decline during school holidays is seen in the school aged (5-19 years) population, with the declines in the adult and infant populations being smaller and following a week later.

**Conclusions**: Given the significant and rapid reductions in incidence, these results have important public health implications. Closure or extension of holiday periods could be an emergency option for state governments.

## Introduction

In 2009, the United Kingdom experienced a summer influenza epidemic, with the National Health Service resorting to pharmacy dispensed oseltamivir to slow the growth. However, when schools closed in August for the six-week summer break, the epidemic dropped to almost zero within a few weeks ^1^. This was an extreme example of an association that has been documented in other countries, though generally for shorter closures ^2– 4^. A 2013 review of both deliberate and non-deliberate school closures concluded that even without co-interventions, closure of schools could reduce influenza transmission during an outbreak ^3, 5^.

The association of school closure and influenza rates has been only sparsely documented in Australia, partly because school holidays generally fall outside the peak influenza period. However, 2019 saw an early epidemic of influenza in Australia with rates around five times normal for the May-July period, with consequent hospitalisation and deaths also increased. Because of the early high rates, winter influenza in 2019 also coincided with the mid-year school breaks, which appeared to be associated with a dip in influenza incidence. Since each country’s school system differs in features such as age range, classroom structure and sizes, and class mixing, we thought it important to document the size of any impact in Australia. Furthermore, the variation by states, and the ability to examine delay to changes in the non-school population would extend the range of sub-questions we could address.

To explore any relationship between the school holiday closure and influenza cases, we examined the relationship between changes in influenza incidence with the school holiday closure dates in 2019 using the different holiday dates that apply in the Australian states and territories.

## Methods

### Setting

Influenza is a notifiable disease in all Australian states and territories ^6^. We collected influenza data notified weekly to state and territory health departments, for the period of week 19 (mid-May) to week 39 (early October) 2019, which corresponds to the influenza season in Australia.

We included data from five states (New South Wales, Queensland, Victoria, South Australia, Western Australia) and one territory (Australian Capital Territory); we excluded data from one state (Tasmania) and one territory (Northern Territory) due to paucity of available data and small population sizes.

### Data sources and extraction

States and territories differ in how they collect and report data on influenza cases. New South Wales ^7^ and Queensland ^8^ report the number of samples that test positive for Influenza A and B (lab-confirmed); Australian Capital Territory ^9^ reports the number of influenza (lab) notifications to the state of influenza A, influenza B, and co-infections of influenza A and B; whilst Western Australia ^10^, Victoria ^11^ and South Australia ^12^ report the number of lab-confirmed influenza cases (the specific strains are unspecified).

We collected influenza data as it was reported by each jurisdiction. We collected data on public school holiday closures between week 19 and week 39 from each state or territory’s education department website.

Weekly, numerical influenza data were available from reports produced by the Health Departments in New South Wales, Queensland, Western Australia, Victoria, and Australian Capital Territory. For South Australia, the number of lab-confirmed influenza cases for weeks 19–24 were extracted from a figure (using webplotdigitiser ^13^) since weekly numerical data were available only for weeks 25–35. Data on school holidays were extracted from each state or territory’s website. Raw data are provided (see *Data availability* section, Appendix 1) ^14^.

### Statistical methods

Weekly counts of influenza cases were collected from five states and one territory across Australia for the first 10 months of 2019. We restricted analysis to the period from week 19 (beginning of school term 2) to week 39 of 2019 (end of school term 3). Due to differences in the population numbers of each state and territory, differences in the methods of data collection (e.g. some states use only sentinel sites), and insufficient number of states to reliably fit a random effects model, the analysis was conducted separately by state.

We used segmented regression ^15^ to model the weekly counts and specified a negative binomial distribution to account for overdispersion. Explanatory factors included the linear effect of week (slope), initial effect of holiday (change in level), change in linear effect of week after start of holiday (change in slope), initial effect of returning to school, and change in linear effect of week after school return (Appendix 2) ^16^. Goodness-of-fit of the models was assessed by plots of observed versus expected counts, plots of residuals versus fitted values, and Pearson’s Chi-square test. The main exposure for our analysis was the July school holiday period of two weeks. Due to the delay from exposure to the virus to confirmation of infection status, we included a lag of one week ^17^. The effects are reported as a % change in level (with 95% confidence interval) as well as a percentage change in slope (with 95% confidence interval). We hypothesized that the holiday period (lagged by one week) would lead to reductions in weekly counts of influenza cases.

Statistical analysis was conducted using SAS University Edition 9.4 for Windows.

## Results

**Figure 1.**
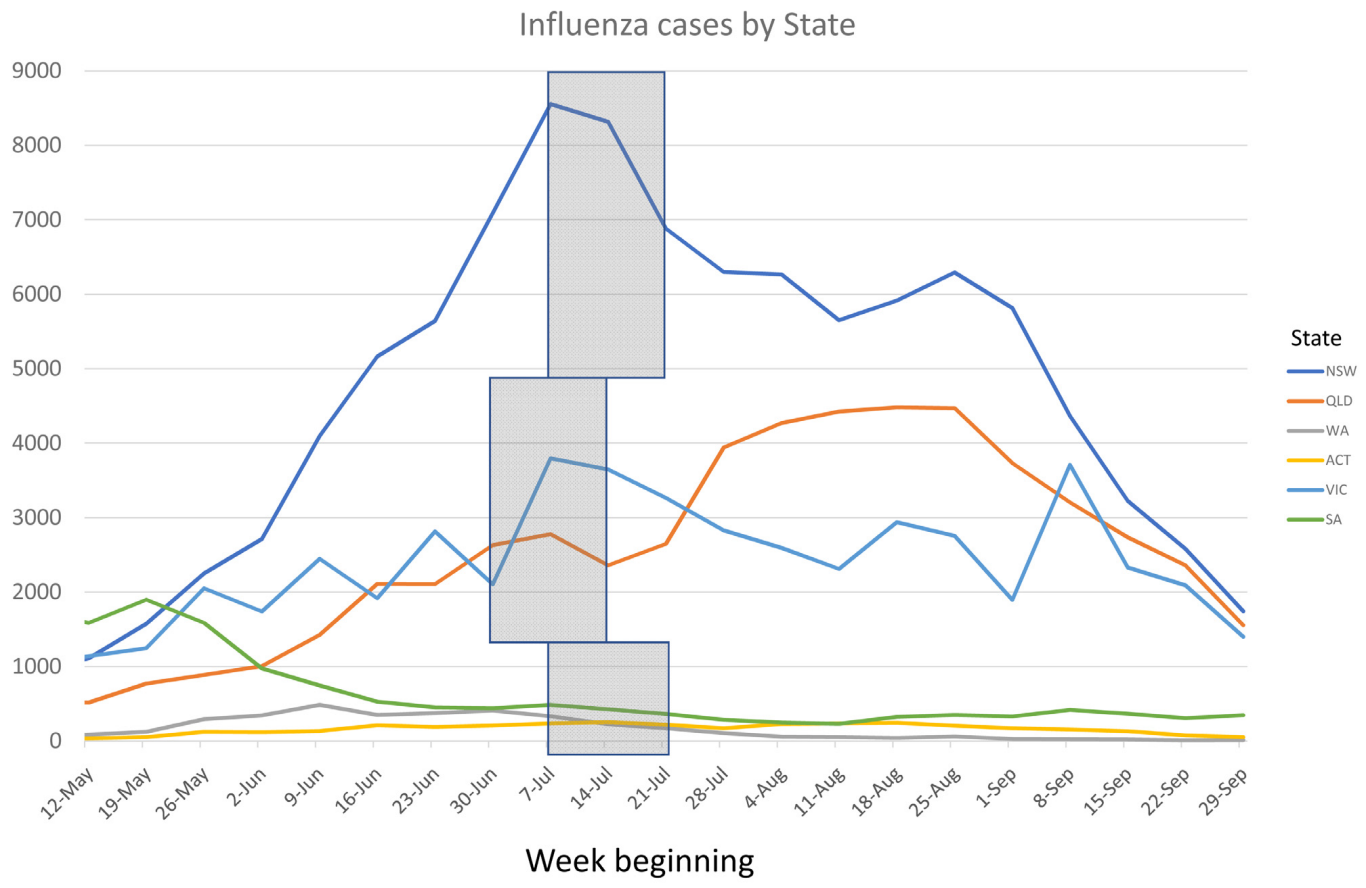
Rates of influenza for the included Australian states and territory (boxes show school holidays periods). NSW, New South Wales; QLD, Queensland; WA, Western Australia; ACT, Australian Capital Territory; VIC, Victoria; SA, Southern Australia.

Figure 1 Influenza cases for the included states and territory Figures for each individual state or territory are provided in Appendix 3 ^16^. 

The estimates for the initial effect and the subsequent slopes ( Table 1) show significant declines for all states except South Australia, which had already passed its peak by the time of the school holidays, and Victoria The models showed acceptable goodness-of-fit (Appendix 3 and 4) with Pearson’s Chi-square tests all indicating insufficient evidence of lack of fit (P>0.05).

**Table 1.** Estimates (95% CI) from segmented regression models of weekly influenza counts.

State	Pre-holiday linear effect of week	Change in level due to school holiday *	Change in slope due to holiday *	Change in level after school return ^	Change in slope after school return ^
NSW	**+26%** (+22%, +31%); P<.0001	**-50%** (-66%, -27%); P=.0004	**-27%** (-53%, +13%); P=.15	**+38%** (-33%, +185%); P=.38	**-6%** (-40%, +46%); P=.78
QLD	**+24%** (+19%, +29%); P<.0001	**-41%** (-59%, -16%); P=.004	**-10%** (-40%, +36%); P=.63	**+79%** (-8%, +150%); P=.087	**-19%** (-46%, +22%); P=.31
VIC	**+13%** (+7%, +18%); P<.0001	**-2%** (-39%, +57%); P=.93	**-21%** (-54%, +36%); P=.40	**+1%** (-58%, +143%); P=.98	**+7%** (-37%, +84%); P=.80
WA	**+10%** (+1%, +19%); P=.03	**-65%** (-84%, -21%); P=.012	**-44%** (-88%, +41%); P=.22	**+7%** (-77%, +390%); P=.93	**+32%** (-48%, +231%); P=.56
SA	**-17%** (-20%, -14%); P<.0001	**+28%** (-14%, +90%); P=.22	**-6%** (-41%, +51%); P=.81	**+21%** (-44%, +163%); P=.63	**+33%** (-17%, +114%); P=.23
ACT	**+20%** (+14%, +26%); P<.0001	**-42%** (-64%, -6%); P=.025	**-35%** (-62%, +13%); P=.13	**+125%** (-9%, +455%); P=.079	**+8%** (-38%, +87%); P=.79

*Compared to pre-holiday period ^Compared to holiday period NSW, New South Wales; QLD, Queensland; VIC, Victoria; WA, Western Australia; SA, South Australia; ACT, Australian Capital Territory.

Results shown in the table can be interpreted as follows, using NSW as an example: In NSW prior to the school holiday the rate of influenza cases was increasing by 26% (+22%, +31%) per week. The school holiday was associated with a 50% decrease (-27%, -66%) in level as well as a 27% decrease (-53%, +13%) in slope. After the students returned to school, there was a 38% increase (-33%, +185% increase) in level and 6% decrease (-40%, +46%) in slope. QLD, ACT and WA showed a similar pattern whereas school holiday appeared to have little impact in SA and VIC.

**Figure 2.**
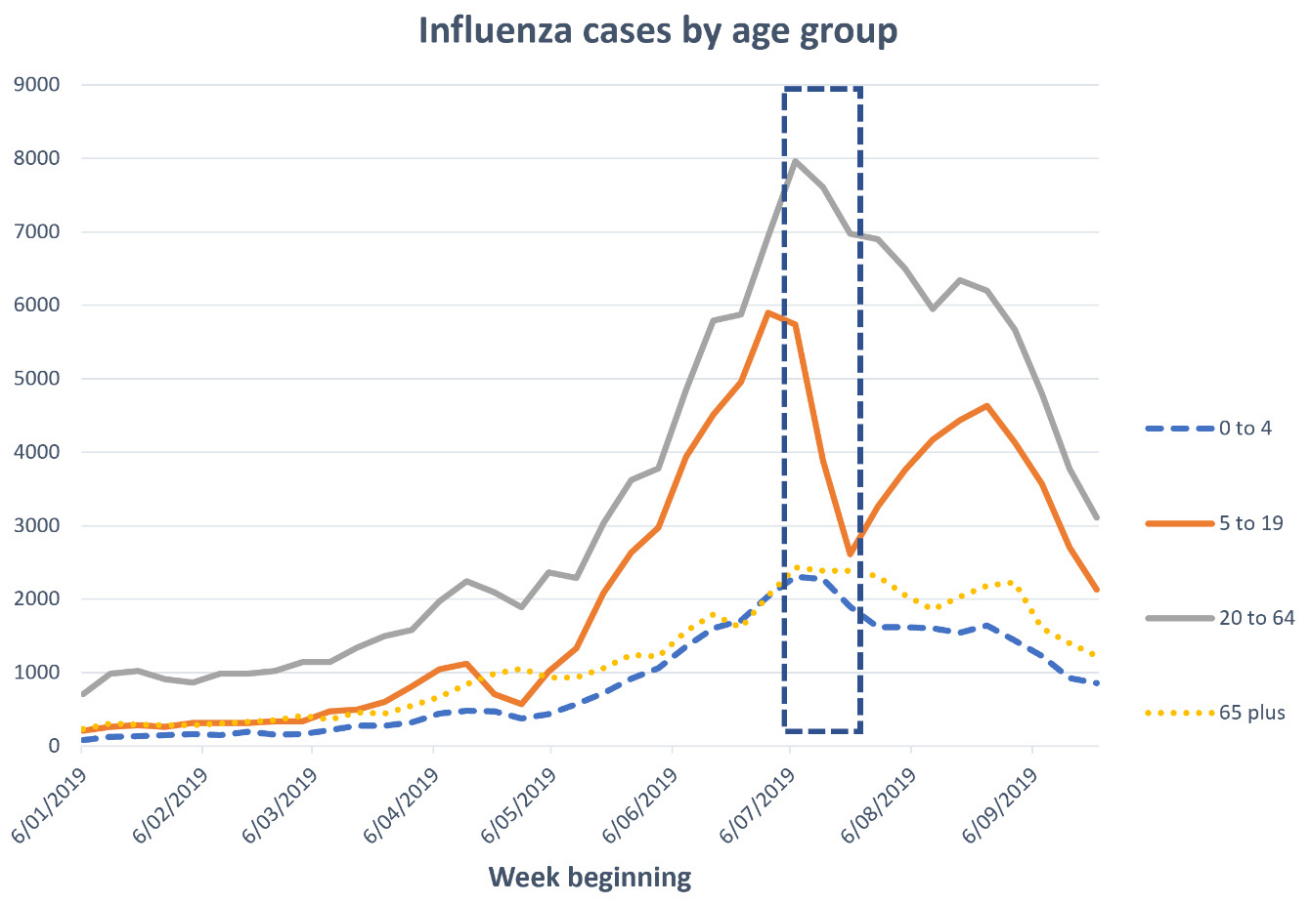
Influenza cases by age group (box shows the school holidays period).

The data on influenza rates by age group ( Figure 2) show the first decline during school holidays is seen in the school aged (5–19 years) population, with the decline in the adult (20–64 years) population being smaller and following a further week later, and with even smaller and delayed drops in the infant (1–4) and elderly (65+ years) age groups. We did not have access to the state specific age bands, and hence have indicated the school holiday closure dates with the largest population groups.

## Discussion

The 2019 Australia influenza notification data show a significant association between school holidays and declines in influenza incidence in most states in Australia. We also found the earliest and largest declines in influenza incidence were in the school aged group (5–19 years), with still later and smaller declines in the adult group (20–64 years), and least impact on the preschool (0–4 years) and over 65’s.

Our findings are consistent with previous reports ^3^ of school closure for usual holiday periods and emergency closure for epidemics as well as studies of how much worse epidemics would have been if schools had *not* been closed ^18^.

The size of the declines is also consistent with those predicted by models of transmission for school closures ^19^. Some states experienced a rebound within weeks of school restarting whereas others saw a continued decline. We do not have a simple explanation for this difference.

The association of school holiday closure with declines in influenza has several potential explanations besides a causal effect. First, cases of influenza might be underreported because of delayed presentation or non-presentation during the school holiday period, for example, because with parents able to care for them they do not attend for medical certificates. However, if this were true, we would expect to see an immediate restart after school returns, which is not the case. Second, it could be because of other societal changes, such as parents also being on holiday and hence less transmission at work. However, if this were true, we would expect to see a simultaneous reduction in both child and adult cases. Hence, the most likely remaining explanation seems to be causal reduction in transmission from school holidays. However, a final issue is that, even if reduction in incidence is real, it not clear whether there is a net annual decrease or merely a delay in total annual cases.

There are some limitations to both our data and the analysis. Good quality, numerical weekly influenza data were unavailable for all states – e.g., South Australia reports its data as a mix of figures and numbers. This may have introduced some errors into the accuracy of the numbers for those states. We contacted the Australian National Notifiable Diseases Surveillance System, which coordinates the national surveillance of influenza in Australia, to obtain raw data for each state and territory. However, the raw data underlying the notifications for 2019 will not be available for release until July 2020. There is also likely to be differences in the accuracy of different states’ data, due to the different methods of collection. For the analysis, we used a lag of one week to allow for the delay from exposure to the virus to confirmation of infection status ^17^. In a sensitivity analysis we refitted the model without a lag and produced consistent results (Appendix 5, *Extended data*) ^16^. The school holiday only lasted 2 weeks hence our estimates of change in slope are imprecise. We used a lag of 1 week to allow for the delay from exposure to the virus to confirmation of infection status. In a sensitivity analyses we refitted the model without a lag. Finally, we did not include a control group (i.e. previous years’ data), although existing data on notifications of laboratory-confirmed influenza in Australia between 2013 and 2019 shows the notifications in 2019 to be higher than the 5-year average, and higher than all of the years other than 2017 ^20^.

Given the size of the effect, these results have important public health implications as no other intervention may have comparable effects. Hence closure or extension of holiday periods could be an emergency option for state governments. Vaccinating children against influenza has also been shown to substantially reduce influenza attack rates in *both* children and adults ^21^. Thus, in addition to encouraging influenza vaccination, the Centres for Disease Control has a number of suggestions in their guidance, such as encouraging students and staff to stay home when sick, liberalising sickness policies during epidemics, encouraging respiratory etiquette, encouraging hand hygiene, regular cleaning of shared surfaces such as door handles and faucets, and providing a “sick room” to quarantine students with flu-like illnesses ^22^. An additional strategy is to consider face masks, which, with hand hygiene, appear to substantially reduce transmission. All these strategies would need to be triggered by health departments to schools at an appropriate point in an epidemic or pandemic.

## Data availability

### Underlying data

This project contains the following underlying data: Australian state influenza notifications and holiday school closures in 2019: Appendix 1 – Underlying data DOI: 10.26139/5c47ae4cd8e16 ^14^

Data can be accessed at: https://research.bond.edu.au/en/datasets/australian-state-influenza-notifications-and-school-closures-in-2

### Extended data

This project contains the following extended data: Australian state influenza notifications and school holiday closures in 2019: Appendix 2-5 – Extended data DOI: 10.4225/57/555d781f8f2a3 ^16^

Data can be accessed at: https://research.bond.edu.au/en/datasets/australian-state-influenza-notifications-and-school-closures-in-2-2

Data are available under the terms of the Creative Commons Attribution 4.0 International license (CC-BY 4.0).
