# Supplementary material for: Australian state influenza notifications and school holiday closures in 2019
Source: F1000Res. 2021 May 26;8:1899. Originally published 2019 Nov 11. [Version 3] doi: 10.12688/f1000research.21145.3 (PMC8097737; doi:10.12688/f1000research.21145.3)
Supplement: Peer review history

# Peer review: Reviewer response for version 2

**DOI:** 10.5256/f1000research.29863.r83446

**Published:** 2021-05-04

**Authors:** August Wrotek

**Affiliations:** 1 Department of Pediatrics, Centre of Postgraduate Medical Education, Warsaw, Poland

Influenza is one of the most common paediatric global health concerns, and various strategies have been suggested to decrease the spread of the disease. The authors of the study aimed to address the question of the impact of temporary school closing on the epidemics. In 2019, Australia faced an unexpected situation when the beginning of the winter season coincided with holiday school closing. The methodology of the study based upon segmented regression modelling in order to assess the effect of the school closure. The authors showed a strong significant decrease (ranging from 41% to 65%) in 3 out of 5 analysed states and in 1 territory (ranging from 41% to 65%). Of note, a decrease in the incidence was first noticed in the school-aged children, followed by the decrease in adults and infants (yet, to a smaller degree, and observed a week later). That indirectly proves the role of school-aged children as vectors who increase the disease spread. The results of the study may in fact have important public health implications. After the comments of the first two reviewers, the doubts have been cleared, and appropriate corrections have been made. Although, for example, a sentence "as no other intervention has comparable effects" may be somehow controversial (thus might be considered to be deleted), yet the general results and conclusions of the study may resume the “old-fashioned” approach based upon temporary closing of the school in the periods of highest influenza virus activity, especially since “lockdown”-based strategies have been applied globally in the COVID-19 era. Certainly, the strategies to decrease influenza cases based on school closing need to be well documented, but this study indeed expands our knowledge on this topic.

Is the work clearly and accurately presented and does it cite the current literature?

Yes

If applicable, is the statistical analysis and its interpretation appropriate?

Yes

Are all the source data underlying the results available to ensure full reproducibility?

Yes

Is the study design appropriate and is the work technically sound?

Yes

Are the conclusions drawn adequately supported by the results?

Partly

Are sufficient details of methods and analysis provided to allow replication by others?

Yes

Reviewer Expertise:

Paediatrics, infectious diseases, pulmonology, particularly influenza, RSV, epidemiology, air pollution, inflammatory markers.

I confirm that I have read this submission and believe that I have an appropriate level of expertise to confirm that it is of an acceptable scientific standard.

# Response

**Published:** 2021-05-11

**Authors:** Anna Scott

**Affiliations:** Bond University, Australia

Dear Doctor Wrotek,

 Thank you very much for your kind comments.

 Following on your suggestion, we have rephrased the sentence “no other intervention has comparable effects” to “no other intervention may have comparable effects.”

 With warmest wishes,

 Anna Scott, on behalf of the authors

# Peer review: Reviewer response for version 2

**DOI:** 10.5256/f1000research.29863.r72716

**Published:** 2020-10-12

**Authors:** Núria Torner

**Affiliations:** 1 CIBER Epidemiology and Public Health CIBERESP, University of Barcelona, Barcelona, Spain

The author has satisfactorily replied to my queries.

Is the work clearly and accurately presented and does it cite the current literature?

No

If applicable, is the statistical analysis and its interpretation appropriate?

Yes

Are all the source data underlying the results available to ensure full reproducibility?

Yes

Is the study design appropriate and is the work technically sound?

Partly

Are the conclusions drawn adequately supported by the results?

Partly

Are sufficient details of methods and analysis provided to allow replication by others?

Partly

Reviewer Expertise:

Surveillance, prevention and control of infectious disease outbreaks, with specific experience in seasonal influenza.

I confirm that I have read this submission and believe that I have an appropriate level of expertise to confirm that it is of an acceptable scientific standard.

# Peer review: Reviewer response for version 1

**DOI:** 10.5256/f1000research.23276.r66760

**Published:** 2020-07-27

**Authors:** Núria Torner

**Affiliations:** 1 CIBER Epidemiology and Public Health CIBERESP, University of Barcelona, Barcelona, Spain

Seasonal Influenza Surveillance deploying epidemiological and virological data on patients with influenza virus infection is a global task performed in coordination with WHO requirements. These include sentinel surveillance and hospital surveillance for severe cases of laboratory confirmed influenza. Even though influenza has been studied in depth, there are still some issues that need to be addressed, at least to encourage increase in vaccine uptake and recommendations.

 There are several issues that are not clear and being this a point observation of just one season, inference is not sound enough to ascertain as evidence. Comparison with previous seasons would be desirable.

**Comments**:

1. One highly contrasted issue is the amplification that children trigger as to transmission of infection to their siblings and household members from the school setting. And this might be prevented by vaccination. (Basta *et al*. 2009) ^1^. So this is not new but it might be relevant for Australia to implement preventive measures, if so this should be stated.
2. During epidemic periods transmission is high and the number of syndromic cases make laboratory confirmation out of range of heath systems. Is this so in Australia? As stated in data sources only laboratory confirmed cases are taken into account for reporting:
*States and territories differ in how they collect and report data on flu cases. New South Wales* *^7^* *and Queensland* *^8^* *report the number of samples that test positive for Influenza A and B (lab-confirmed); Australian Capital Territory* *^9^* *reports the number of influenza (lab) notifications to the state; and Western Australia* *^10^* *, Victoria* *^11^* *and South Australia* *^12^* *report the number of lab-confirmed influenza cases (strains unspecified*).
  What is the difference between the three (besides type identification)?
  Positive for Influenza A and B (lab-confirmed); number of influenza (lab) notifications; and number of lab-confirmed influenza cases (strains unspecified). Do the authors mean type and subtype by strain? Or does it make reference to the strain type?
  Type A
  Subtype A H1N1
  Strain: A/Brisbane/59/2007 (H1N1)
  Use the correct term.
3. There is also the climate factor to be taken into account. Do all regions included in the study have the same temperature, humidity range? Some differences in decrease of transmission could also be due to a lesser degree of transmission such as in Victoria and Southern Australia for example. Did this particular season have a different climatology that made it coincide with the school holiday period?
4. The results of the pre-school closure period refer to the baseline epidemic burden of disease, if so then there is a decline before school closure? By the way, the term school closure can be misleading as to a containment preventive measure that can be implemented to stop transmission and what is referred to in the study should be specific to school holiday closure. What has happened in other epidemic seasons should be included to be able to compare and strengthen the results obtained.
5. In the introduction first line: A pandemic is global, so it wasn’t just the UK that was affected. As a matter of fact school closure emerged as one of the non-pharmaceutical strategies during that pandemic and has been implemented in the present COVID-19 pandemic. Sentence should be rephrased.
  The authors findings could be in the same line as Eames (2013) ^2^ who stated that allowing some ﬂexibility in holiday timing would provide maximum health beneﬁt from the holiday period, yet this is difficult to carry out because of the different social and labor implications it has for schools, parents, and care givers. Was this the aim of the study?
6. In the conclusions the authors state that: no other intervention has comparable effects. There is no comparison in the study to other non-pharmaceutical mitigation measures. Can you include a reference to this statement?

**Minor comments:**

- Include label on Figure 1, are they number of cases in total for all states or incidence rates? Please complete.
- Why does Fig 1 have dates in the x-axis and Figure 2 the week number? Why different, they should be the same.

Is the work clearly and accurately presented and does it cite the current literature?

No

If applicable, is the statistical analysis and its interpretation appropriate?

Yes

Are all the source data underlying the results available to ensure full reproducibility?

Yes

Is the study design appropriate and is the work technically sound?

Partly

Are the conclusions drawn adequately supported by the results?

Partly

Are sufficient details of methods and analysis provided to allow replication by others?

Partly

Reviewer Expertise:

Surveillance, prevention and control of infectious disease outbreaks, with specific experience in seasonal influenza.

I confirm that I have read this submission and believe that I have an appropriate level of expertise to confirm that it is of an acceptable scientific standard, however I have significant reservations, as outlined above.

## References

[1] : Strategies for pandemic and seasonal influenza vaccination of schoolchildren in the United States. *Am J Epidemiol*.2009;170(6) : 10.1093/aje/kwp237 679-86 10.1093/aje/kwp237 19679750PMC2737588

[2] : The influence of school holiday timing on epidemic impact. *Epidemiol Infect*.2014;142(9) : 10.1017/S0950268813002884 1963-71 10.1017/S0950268813002884 24230961PMC9151328

# Response

**Published:** 2020-09-24

**Authors:** Anna Scott

**Affiliations:** Bond University, Australia

Thank you very much for your comments. We provide our responses below:

1. We agree that a multi-year comparison would be preferable. However, for 2020, the number of flu infections is atypical (because of COVID), and for years prior to 2019, raw data is very difficult to obtain as it requires multiple data access applications to the individual states and to the Commonwealth. We have included the lack of control group as a limitation of the study, and provided a reference to a Government document comparing the trend from 2019 to years going back to 2013.  
2. Thank you for providing this reference. We added the following sentence to the Discussion: "Vaccinating children against influenza has also been shown to substantially reduce influenza attack rates in *both* children and adults (Basta et al 2009)."
3. The relatively small size of the Australian population (approximately 25 million) makes laboratory confirmation a bit easier than in many other, more populous countries (e.g. in Europe, North America, South America). As we note in the methods section, we report lab-confirmed cases.
4. States/territories report influenzas a bit differently from each other. States of NSW and QLD report influenza A and influenza B only. ACT reports influenza A, influenza B, and coinfections with influenza A and B. The remaining states report lab-confirmed influenzas but do not specify strains. We have made this clearer in the text.
5. The temperatures were 1.6 deg above the long-term average, in line with global warming but with no unusual anomaly in July 2019 (http://www.bom.gov.au/climate/mwr/aus/mwr-aus-201907.pdf). We do not have humidity data, but rainfall was lower than usual, suggesting lower humidity.
6. There was no decline before school closure – as Figure 1 shows, the decline only occurs within the grey boxes (the holiday periods). Any apparent decline – e.g. NSW – is because we have joined the dots of the weekly reported numbers.
7. We have modified the title, and edited the body of the manuscript to refer to school holiday closures, to make this clearer.
8. We have replaced the term 'pandemic' with the term 'epidemic' in the first line of the introduction.
9. Eames' study used modelling to estimate a counterfactual: what would have happened if the UK had NOT closed the schools during the 2009 epidemic - and found the epidemic would have been much worse than it was. We estimated what happened with the actual school holiday closure in Australia - and found the influenza incidence decreased. Our study and Eames study are therefore consistent. We have added this point to the discussion section.
10. We added references to the newly updated Cochrane review by Tom Jefferson, on physical interventions to reduce the spread of respiratory viruses.
11. They are cases - we edited the figure and its title to clarify this.
12. We edited the figures so they both refer to dates for consistency.

# Peer review: Reviewer response for version 1

**DOI:** 10.5256/f1000research.23276.r57787

**Published:** 2020-01-16

**Authors:** Shweta Bansal, Casey Zipfel

**Affiliations:** 1 Department of Biology, Georgetown University, Washington, DC, USA

**Summary**:

 Australia’s influenza season does not typically coincide with school holidays. However, in 2019, the influenza season occurred earlier than usual, allowing the opportunity to evaluate the impacts of school closure on influenza dynamics in Australia. In this article, the authors present state and territory-level influenza data by week, and perform segmented regression, comparing influenza incidence before and after the school holiday. They also count weekly increases and decreases before, during, and after the school holiday, and show age-based dynamics. This article asks an important question of public health relevance to non-pharmaceutical interventions in a setting where it has not been previously investigated in a concise manner. However, this article could be strengthened through additional framing, increased statistical rigor, and more nuanced interpretation.

**Comments**:

- The introduction leaves several unanswered questions that would help to more clearly identify the importance of the study. The relationship between school closure and influenza dynamics has been explored in a number of previous papers, with both epidemiological data and mechanistic models. Thus, it is unclear to me why is it important to consider this relationship in Australia, specifically. Do the authors have hypotheses regarding trends or mechanisms that might be different from other countries? Is it important for public health planning? This question seems especially salient to me since school closures and the influenza season in Australia do not typically coincide, so these epidemiological trends may not be typical for Australia.
- The article concludes that there is a significant reduction in influenza in most states in Australia based on the results of segmented regression comparing influenza incidence level and slope before and after the holiday period. I am not totally convinced by this conclusion, as Table 1 shows that the change in level is not significant for Victoria, Australian Capital Territory, and Southern Australia, and the change in slope is not significant for Queensland, Victoria, Western Australia, and Southern Australia. This is contrary to the section of the results that states that “the estimates for the initial effect and the subsequent slope show significant declines for all states except South Australia”.  These results could be more carefully interpreted. This would give rise to an interesting question: what are the differences between the states/territories that result in different dynamics?
- I think this article could be strengthened with a more robust time series modeling approach, like ARIMA modeling. This would be particularly useful because the predicted vs. residual plot in Appendix 4 appears to indicate heteroskedasticity in the data. Including model terms in a time series model could handle this issue, as well as temporal autocorrelation.
- The results also provide the percent of weeks in the pre-vacation, during-vacation, and post-vacation period in which there is a weekly decrease in influenza incidence, indicating that there is a higher proportion of decreases in the during and post-vacation period. However, the pre-vacation period seems to include all of the early part of the season. If there were numerous decreases in this time period, then the onset of the influenza season would not have occurred. Thus, this finding may be a bit circular.
- It would be useful to compare this influenza season, which co-occurs with the holiday period, with another influenza season that does not co-occur with the holiday period, as a control. This could help to isolate the impact of school closure on influenza dynamics.
- The authors specify mid-May to early September as their study period. However, it’s unclear what total population and age-specific dynamics looked like post week 35. Past studies have shown that school holidays simply delay dynamics so that the epidemic wave recovers to pre-holiday rates after the holiday. I would be curious if this is the case here.
- In the model, a one-week lag was assumed. Is there evidence about care-seeking and influenza surveillance/reporting to support this? The discussion mentions a sensitivity analysis performed with no lag, but I wonder if the lag could be longer due to a combination of delay to seeking healthcare and delay in reporting.
- The fourth paragraph of the discussion comments on the possible causes of the association between school closure and influenza. This paragraph rules out several possible explanations but does not then provide a plausible explanation.

**Minor comments:**

- A y-axis label on Figure 1 and Figure 2 would be helpful. The Figure 1 caption says it is the influenza rate, so I assume it is influenza cases per week, but this could be clearer.
- Is the x-axis in Figure 2 the week number? It would be easier to interpret if it was put into the same units as Figure 1, with dates instead of week counts.
- Does Figure 2 include cases for all of the states and territories combined?
- In Table 1, it is unclear whether the changes described (in both slope and level) are comparing the during-vacation period or the post-vacation period to the pre-vacation period. The observed and predicted plots in Appendix 3 appear to show 3 different segments fit for each of these periods, but it is unclear what change is reported in Table 1.

Is the work clearly and accurately presented and does it cite the current literature?

Partly

If applicable, is the statistical analysis and its interpretation appropriate?

Partly

Are all the source data underlying the results available to ensure full reproducibility?

Yes

Is the study design appropriate and is the work technically sound?

Partly

Are the conclusions drawn adequately supported by the results?

Yes

Are sufficient details of methods and analysis provided to allow replication by others?

Yes

Reviewer Expertise:

Modeling social and spatio-temporal dynamics of infectious disease, with specific experience in seasonal influenza.

We confirm that we have read this submission and believe that we have an appropriate level of expertise to confirm that it is of an acceptable scientific standard, however we have significant reservations, as outlined above.

# Response

**Published:** 2020-09-24

**Authors:** Anna Scott

**Affiliations:** Bond University, Australia

Thank you very much for your comments. We provide our responses to each comment below:

1. We have added to the introduction the following: "Since each country’s school system differs in features such as age range, classroom structure and sizes, and class mixing, we thought it important to document the size of any impact in Australia. Furthermore, the variation by states, and the ability to examine delay to changes in the non-school population would extend the range of sub-questions we could address."
2. We agree, and have revised our interpretation of the results.
3. We are unfamiliar with ARIMA modelling, but generally prefer simpler approaches. Our main purpose for the statistical analysis is to test hypotheses for each state on whether the school closures have resulted in changes in levels and/or changes in slopes with regards to influenza cases. The goodness-of-fit measures suggest the regression models provide adequate fit hence we believe our results are valid.
4. We agree, and have removed this analysis from the manuscript.
5. Unfortunately we were unable to formally compare as the previous years’ raw numbers are not readily available. We have included the lack of control group as a limitation of the study, and provided a reference to a Government document comparing the trend from 2019 to years going back to 2013.  
6. We have included 1 additional month of data – up until the start of the October school holiday period.
7. A lag of 2 weeks would only be plausible if a person being exposed to influenza on the last week of school prior to the vacation would generally be reported at least 2 weeks and 3 days later. Given “symptoms can begin about 2 days (but can range from 1 to 4 days) after the virus enters the body” this delay seems unrealistic.
8. We agree, and have added the following: "Hence, the most likely remaining explanation seems to be causal reduction in transmission from school holidays."
9. Comments: 9-12. We have revised the Figures and Tables to address these comments.
